# Effects of prolonged incubation period and centralized quarantine on the COVID-19 outbreak in Shijiazhuang, China: a modeling study

**DOI:** 10.1186/s12916-021-02178-z

**Published:** 2021-12-07

**Authors:** Wenlong Zhu, Mengxi Zhang, Jinhua Pan, Ye Yao, Weibing Wang

**Affiliations:** 1 School of Public Health, Shanghai Institute of Infectious Disease and Biosecurity, Fudan University, 138 Yi Xue Yuan Road, Shanghai, 200032 China; 2 Key Laboratory of Public Health Safety of Ministry of Education, Fudan University, 138 Yi Xue Yuan Road, Shanghai, 200032 China; 3 Department of Epidemiology, School of Public Health; Shanghai Institute of Infectious Disease and Biosecurity; Key Laboratory of Public Health Safety (Ministry of Education), Fudan University, 138 Yi Xue Yuan Road, Shanghai, 200032 China

**Keywords:** COVID-19, Epidemiology, Incubation period, Non-pharmaceutical intervention, SEIR model

## Abstract

**Background:**

From 2 January to 14 February 2021, a local outbreak of COVID-19 occurred in Shijiazhuang, the capital city of Hebei Province, with a population of 10 million. We analyzed the characteristics of the local outbreak of COVID-19 in Shijiazhuang and evaluated the effects of serial interventions.

**Methods:**

Publicly available data, which included age, sex, date of diagnosis, and other patient information, were used to analyze the epidemiological characteristics of the COVID-19 outbreak in Shijiazhuang. The maximum likelihood method and Hamiltonian Monte Carlo method were used to estimate the serial interval and incubation period, respectively. The impact of incubation period and different interventions were simulated using a well-fitted SEIR^+q^ model.

**Results:**

From 2 January to 14 February 2021, there were 869 patients with symptomatic COVID-19 in Shijiazhuang, and most cases (89.6%) were confirmed before 20 January. Overall, 40.2% of the cases were male, 16.3% were aged 0 to 19 years, and 21.9% were initially diagnosed as asymptomatic but then became symptomatic. The estimated incubation period was 11.6 days (95% CI 10.6, 12.7 days) and the estimated serial interval was 6.6 days (0.025^th^, 0.975^th^: 0.6, 20.0 days). The results of the SEIR^+q^ model indicated that a longer incubation period led to a longer epidemic period. If the comprehensive quarantine measures were reduced by 10%, then the nucleic acid testing would need to increase by 20% or more to minimize the cumulative number of cases.

**Conclusions:**

Incubation period was longer than serial interval suggested that more secondary transmission may occur before symptoms onset. The long incubation period made it necessary to extend the isolation period to control the outbreak. Timely contact tracing and implementation of a centralized quarantine quickly contained this epidemic in Shijiazhuang. Large-scale nucleic acid testing also helped to identify cases and reduce virus transmission.

**Supplementary Information:**

The online version contains supplementary material available at 10.1186/s12916-021-02178-z.

## Background

Coronavirus disease 2019 (COVID-19) emerged in Wuhan, Hubei Province, China during early December 2019 [1]. The causative virus, SARS-CoV-2, could be spread by asymptomatic, presymptomatic, and symptomatic patients *via* droplets during close face-to face contact [2]. As of 27 April 2021, there were more than 147 million confirmed cases and more than 3 million deaths worldwide [3]. China had 103,503 confirmed cases [3], about half of which were reported in Wuhan during the first wave of the pandemic.

Because of serial strict non-pharmaceutical interventions (NPIs) [4], the first wave of the COVID-19 pandemic in China was controlled by the end of March 2020. However, increasing travels among China and foreign countries and rising infected cases in foreign countries increased the spread of infection, and there were many COVID-19 outbreaks elsewhere in China. The increase in domestic population mobility and relaxation of NPIs had also contributed to local outbreaks. Since the end of March 2020, there were reports of sporadic and localized outbreaks in some important international transportation hub cities and in cities with large populations, such as Beijing [5], Urumqi [6], Dalian [7], Qingdao, Kashgar, and Shanghai. Based on the initial success in controlling the first wave of the pandemic in Wuhan, the introduction of non-pharmaceutical interventions (detailed and rapid close contact tracing; centralized quarantine/isolation; home quarantine; managed closing of communities; traffic restrictions) helped to prevent virus transmission during these subsequent local outbreaks. Moreover, mass nucleic acid testing was implemented to help control these outbreaks. In addition, the virus has mutated because of the global nature of the pandemic; before the herd immunity is established, NPIs continue to play significant roles in controlling this global pandemic.

Shijiazhuang, the capital of Hebei Province, is located in North China and has a population of 10 million and consists of 22 administrative districts. On 2 January 2021, a local outbreak of COVID-19 occurred in Shijiazhuang [8]. This outbreak differed in some ways from previous localized outbreaks. Analysis of the COVID-19 outbreak in Shijiazhuang may therefore be useful as a reference for controlling local outbreaks of COVID-19 in other cities and provide important new information about this disease.

We used publicly available data to analyze the characteristics of the local outbreak of COVID-19 in Shijiazhuang. In particular, we estimated the incubation period and serial interval, used the SEIR^+q^ model to simulate the spread and transmission of COVID-19, and assessed the impact of different non-pharmaceutical interventions.

## Methods

### Data collection

Data on the characteristics of patients with symptomatic COVID-19 in Shijiazhuang from 2 January to 14 February 2021 were from daily reports released by the Health Commission of Hebei Province (http://wsjkw.hebei.gov.cn/). These publicly available data included sex, age, district of residence, and information on several key epidemiological time points (dates of confirmed diagnosis, quarantine/isolation, and first positive nucleic acid test). These data also included information on family relationships among cases. From such information, we could identify whether the cases were from the same family. All data were extracted and entered into a structured database. The inclusion and exclusion criteria for the data collection are as following.

#### Inclusion criteria

COVID-19 symptomatic cases reported during 2 January to 14 February 2021 in Shijiazhuang were included.

Cases who were diagnosed as asymptomatic firstly and then show the relevant symptoms were also included.

All cases were diagnosed according to the Diagnosis and Treatment Protocol for Coronavirus Pneumonia (Trial Version 8).

#### Exclusion criteria

Infected persons with the absence of relevant information (age, sex, district of residence, etc.) were not included. Thus, due to the lack of relevant information, infected patients who were diagnosed as asymptomatic and did not show symptoms later (namely true asymptomatic infections) were not included.

### Case definition

Cases were diagnosed according to the Diagnosis and Treatment Protocol for Coronavirus Pneumonia (Trial Version 8) from the National Health Commission of China. Clinical confirmed diagnosis refers to the symptomatic persons according to CT results and clinical symptoms after the first positive nucleic acid test. When an individual was tested with positive nucleic acid, he/she would be reported to health authorities but need to be confirmed by clinical diagnosis (ground-glass opacities in CT manifestation, consolidative opacity, clinical symptoms, etc.) to become a clinical confirmed case; otherwise, he/she would be categorized to an asymptomatic case. Based on the information from the daily epidemic reports, all confirmed symptomatic cases were classified as “immediately confirmed cases” or “later becoming symptomatic cases”. Immediately confirmed cases were those who were diagnosed soon after or at the same time as symptom onset. Later becoming symptomatic cases were those who were diagnosed with asymptomatic infection during the incubation period and subsequently developed symptoms. A “family cluster” was defined by two or more cases that were family members who lived together (such as parents and children), or as relatives who had contact before diagnosis.

### Four stages of the implementation of interventions

To control the COVID-19 outbreak in Shijiazhuang, serial non-pharmacological interventions (NPIs) were implemented, such as lockdown of the city, suspension of public transportation, building of centralized isolation apartments, and nucleic acid testing for the whole city. The implementation of control activities was divided into four stages (stage 1: 2–5 January; stage 2: 6–9 January; stage 3: 10–19 January; stage 4: 20 January–14 February) based on the start dates of the three rounds nucleic acid testing.

During stage 1, interventions were mainly implemented in Zengcun, a town in Gaocheng District, in which the first case was reported on 2 January. On 3 January, massive nucleic acid testing was performed in Zengcun. On 5 January, the Xiaoguozhuang Village of Zengcun was officially closed.

During stage 2, the first-round of city-wide nucleic acid testing was implemented. On 6 January, serial interventions were performed in Shijiazhuang, such as school closure, suspension of public transportation, and cancellation of all public events. On 8 January, all residents were asked to stay home for 7 days (i.e., the lockdown).

During stage 3, the second-round of city-wide nucleic acid testing began in some residential communities on 10 January and ended on 14 January. To prevent environment-to-human-transmission, more than 20,000 residents of 12 villages in Zengcun Town, Gaocheng District left their homes and were placed in centralized quarantine at another location on 11 January (“distant centralized quarantine”), and these 12 villages were thoroughly disinfected. The residents were allowed to return to villages when the outbreak was under control. The construction of a centralized isolation place the Huangzhuang Apartment began on 14 January, and the first batch of houses was delivered on 17 January. On 15 January, the government declared that the lockdown would be extended to 20 January.

During stage 4, the third round of city-wide nucleic acid testing was performed from 20 to 22 January, during which home quarantine was maintained.

### Statistical analysis

All confirmed symptomatic cases (including immediately confirmed cases and later becoming symptomatic cases) were included in the statistical analysis. The epidemic curves by the date of confirmed diagnoses and the dates of positive nucleic acid testing, the geographical distribution of patients in Shijiazhuang and Gaocheng District were plotted. A *t*-test was used to analyze the difference in the mean age of immediately confirmed cases and later becoming symptomatic cases. A *χ*^2^ test or Fisher’s exact test was used to compare other characteristics of these two groups.

Based on family cluster data, in which the transmission events and the interval between symptom onset could be clearly identified, the best gamma distribution of the serial interval was estimated using the maximum likelihood method. Within a family cluster, a case who developed symptoms 1 to 3 days after the date of symptom onset of the index case may be infected by an unidentified infector [9]. For sensitivity analyses, clusters with delayed detections of 0, 1, 2, and 3 days from the date of symptom onset between index and consecutive generations of cases were used to estimate the serial interval.

To estimate the incubation period, COVID-19 cases with clearly defined periods of possible exposure and date of symptom onset were selected. The period of possible exposure was defined as the days between the earliest possible exposure and the latest exposure. Three parametric distributions (Weibull, Gamma, and Lognormal) were used with the Hamiltonian Monte Carlo method for Bayesian inference to estimate the incubation period [10]. The Leave-one-out Information Criterion (LooIc) was used to evaluate the performance of the three models.

The geographical distribution of COVID-19 cases was presented using ArcGIS software version 10.5 (Environmental Systems Research Institute, Inc.). The statistical analysis and estimations of the serial interval and incubation period were performed with the *rstan* [11] and *MASS* [12] packages in R project version 4.0.2 [13].

### SEIR^+q^ model

**Fig. 1:**
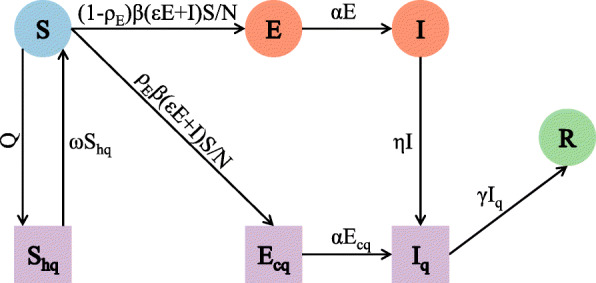
Flow patterns between different compartments in the SEIR^+q^ model. *Q* is the number of comprehensive quarantined persons per day; *ω* is the rate of release from isolation; *ρ*_E_ is the probability of an exposed person being found and centralized quarantine; *β* is the average number of infected individuals per day; *ε* is the transmission coefficient of exposed people (compared with symptomatic people); *α* is the transition rate from latent infection to symptomatic infection; *η* is the isolation/confirmed diagnosis rate of symptomatic people; and *γ* is the transition rate from disease confirmation to recovery or death

To assess the impact of the changes in the incubation period and the influence of serial interventions on the COVID-19 outbreak of Shijiazhuang, the classical compartmental SEIR model (susceptible, exposed, infectious, and recovered) was extended to the SEIR^+q^ model (Fig. 1), which contained three additional compartments (S_hq_, home quarantined susceptible; E_cq_, centralized quarantined exposed; and I_q_, isolated infectious), as described in the equations below and selected initial values (Additional file 1: Table S1) [8, 14–19]. The maximum likelihood method was used to estimate the two unknown parameters (*Q*, number of comprehensive quarantined persons per day; *β*, transmission velocity, number of susceptible persons infected by an infector per day).

$$ \frac{dS}{dt}=\omega {S}_q-Q-\frac{\rho_E\beta \left(\varepsilon E+I\right)S}{N}-\frac{\left(1-{\rho}_E\right)\beta \left(\varepsilon E+I\right)S}{N} $$

$$ \frac{dS_{hq}}{dt}=Q-\omega {S}_{hq} $$

$$ \frac{dE}{dt}=\frac{\left(1-{\rho}_E\right)\beta \left(\varepsilon E+I\right)S}{N}-\alpha E $$

$$ \frac{dE_{cq}}{dt}=\frac{\rho_E\beta \left(\varepsilon E+I\right)S}{N}-\alpha {E}_{cq} $$

$$ \frac{dI}{dt}=\alpha E-\eta I $$

$$ \frac{d{I}_q}{dt}=\alpha {E}_{cq}+\eta I-\gamma {I}_q $$

$$ \frac{dR}{dt}=\gamma {I}_q $$

The fitted model was used to simulate the effects of changes of four key parameters that correspond to different interventions (*Q*, the number of comprehensive quarantined persons per day; 1/*α*, incubation period; 1/*ω*, isolation period; *ρ*_E_, probability of an exposed person being found and centralized quarantined) on the COVID-19 outbreak. The comprehensive quarantine measures (Q), which mainly influenced the number of quarantined susceptible people, included close contact tracking, home quarantine, and similar quarantine measures. The effectiveness of nucleic acid testing (*ρ*_E_), which had a significant impact on the centralized quarantine of exposed people, was a function of the scale and speed of this test. Estimation of unknown parameters and simulation of different scenarios were conducted in R project version 4.0.2 using the *deSolve* package [20].

## Results

**Fig. 2:**
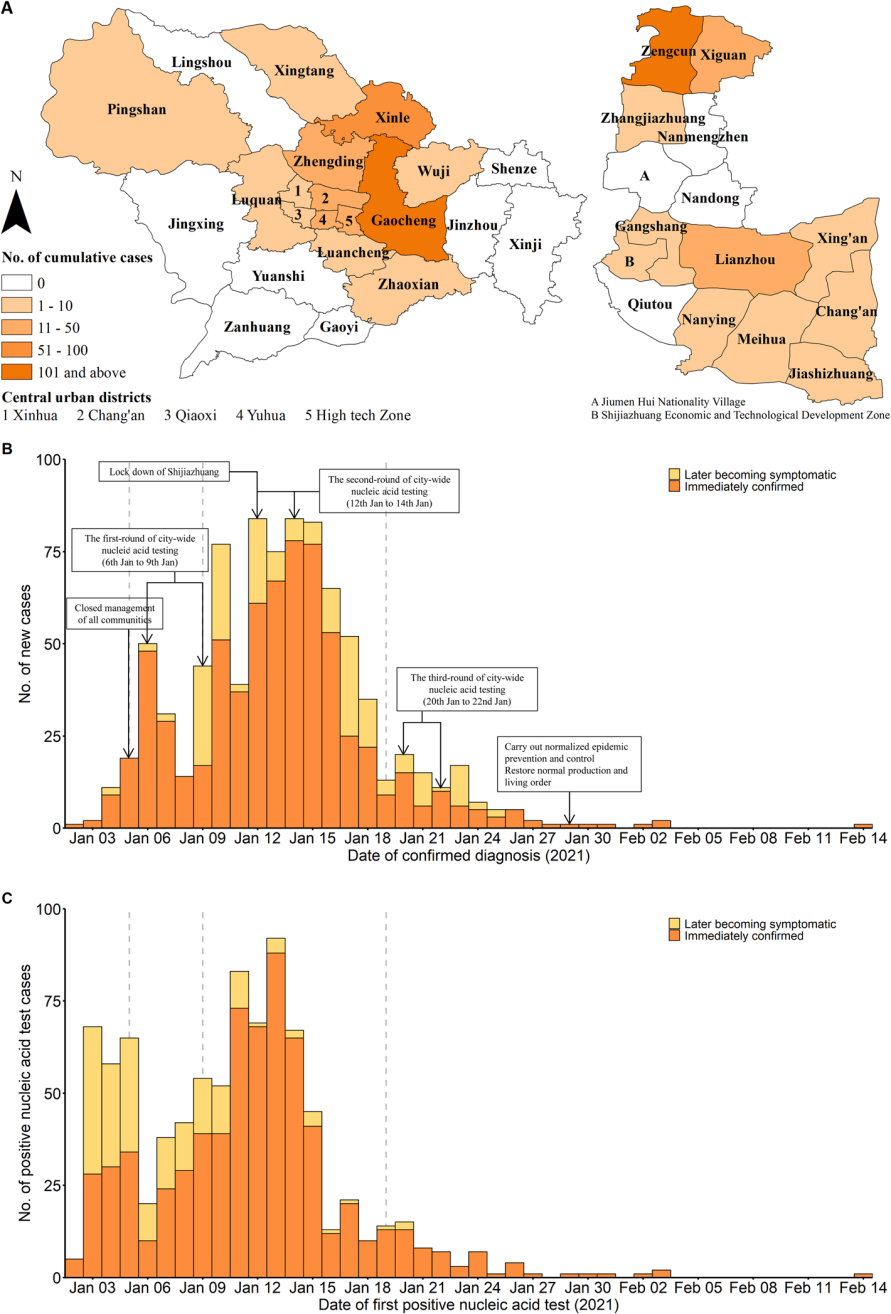
The geographical distribution and epidemic curve of confirmed COVID-19 cases in Shijiazhuang. Geographical distribution of confirmed COVID-19 cases in Shijiazhuang (left, **A**) and Gaocheng District (right; **A**). Number of confirmed diagnosis cases (**B**) and number of positive nucleic tests (**C**) in Shijiazhuang on different dates

From 2 January to 14 February 2021, there were 869 patients with symptomatic COVID-19 in the 14 districts of Shijiazhuang (there were no cases in 8 districts), 80.7% of these patients were from Gaocheng District, and 90.9% of the patients from Gaocheng District lived in Zengcun (Fig. 2A). Among all 869 patients, 78.1% were immediately confirmed cases and 21.9% were initially asymptomatic but subsequently classified as symptomatic (later becoming symptomatic cases), 40.2% were male, the median age was 47.0 years, most patients (81.2%) were 20 to 79 years-old, and 47.8% were centrally quarantined before diagnosis (Table 1). There was no significant difference in sex between the immediately confirmed cases and later becoming symptomatic cases. However, the later becoming symptomatic cases were younger (*P* < 0.001) and a greater percentage of immediately confirmed cases were centrally isolated (*P* < 0.001).

**Table 1:** Characteristics of patients with laboratory-confirmed COVID-19 in Shijiazhuang^a^

Characteristic		Overall	Immediately confirmed cases	Later becoming symptomatic cases	***P*** value
Patients		869 (100.0)	679 (78.1)	190 (21.9)	
**Sex**	Male	349 (40.2)	265 (39.1)	84 (44.2)	0.234
	Female	519 (59.8)	413 (60.9)	106 (55.8)	
**Age (years)**		47.0 (30.0,60.0)	47.0 (31.0,60.0)	45.0 (24.5,59.8)	**<0.001**
**Age group (years)**	0–19	142 (16.3)	99 (14.6)	43 (22.6)	0.055^†^
	20–39	231 (26.6)	188 (27.7)	43 (22.6)	
	40–59	272 (31.3)	216 (31.8)	56 (29.5)	
	60–79	203 (23.4)	159 (23.4)	44 (23.2)	
	80+	21 (2.4)	17 (2.5)	4 (2.1)	
**Centralized quarantine**	No	454 (52.2)	309 (45.5)	145 (76.3)	**<0.001**
	Yes	415 (47.8)	370 (54.5)	45 (23.7)	
**Cases per period**	2–5 Jan	33 (3.8)	31 (4.6)	2 (1.1)	**0.007**
	6–9 Jan	139 (16.0)	108 (15.9)	31 (16.3)	
	10–19 Jan	607 (69.9)	480 (70.7)	127 (66.8)	
	20 Jan–14 Feb	90 (10.4)	60 (8.8)	30 (15.8)	

^a^ All values are given as N (%) or median (IQR). ^†^ *P* value calculated by χ^2^ test after merging 60-79 and 80+ groups

Most (89.6%) COVID-19 patients were confirmed before 20 January, namely, before the third-round of the city-wide nucleic acid testing (Table 1, Fig. 2B). However, more of the later becoming symptomatic cases were clinical diagnosed after 20 January (*P* = 0.007). As a result of the first and second rounds of city-wide nucleic acid testing, most patients (93.9%) were identified and isolated before 20 January (Fig. 2C), leading to timely control of the source of infection. For all patients, the mean lag time from a positive nucleic acid test to clinical confirmation (namely make a definite diagnosis, which was used for the distinguish of asymptomatic cases and later becoming symptomatic cases) was 2.7 days. Notably, the mean lag time from a positive nucleic acid test to clinical confirmation was longer for later becoming symptomatic cases than immediately confirmed cases (7.7 days *vs.* 1.3 days, *P* < 0.001).

**Fig. 3:**
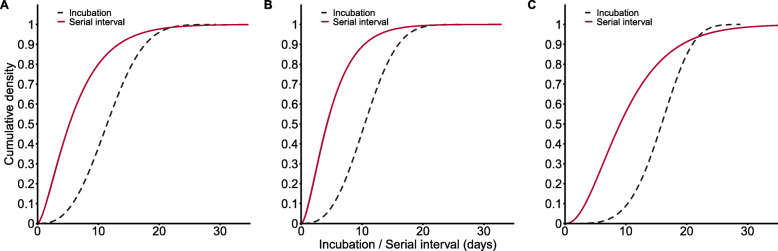
Cumulative density functions of the estimated incubation period (Weibull distribution) and serial interval (Gamma distribution) of COVID-19 cases in Shijiazhuang for all cases (**A**), immediately confirmed cases (**B**), and later becoming symptomatic cases (**C**)

**Fig. 4:**
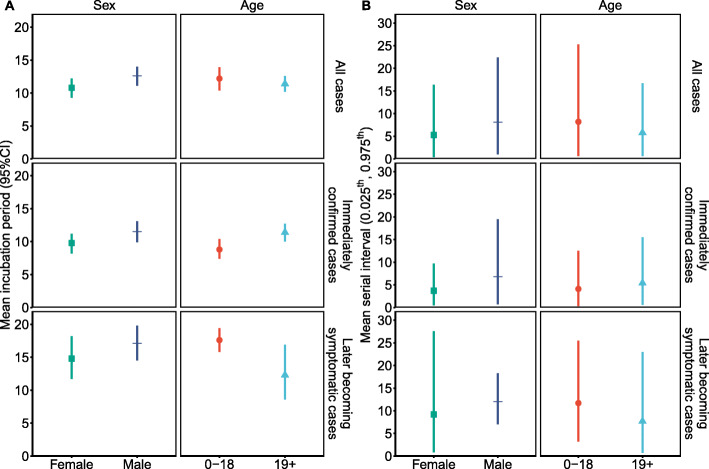
The estimated incubation period (**A**) and serial interval (**B**) for COVID-19 patients stratified by sex and age. Incubation period was presented by mean and 95% confidence interval (95% CI). Serial interval was presented by mean and 0.025^th^, 0.975^th^ percentiles (0.025^th^, 0.975^th^)

According to the LooIc criterion, the Weibull distribution provided the best fits to data for all cases, immediately confirmed cases, and later becoming symptomatic cases (Additional file 1: Table S2). The average incubation period was 11.6 days (95% CI 10.6, 12.7 days) for all cases, 10.6 days (95% CI 9.5, 11.7 days) for immediately confirmed cases, and 15.8 days (95% CI 13.7, 17.8 days) for later becoming symptomatic cases (Table 2). We also analyzed the time between symptom onset in 74 consecutive generations of patients and 64 corresponding index patients, with exclusion of those who reported the same date of symptom onset as the index patients. The serial intervals of all cases, immediately confirmed cases, and later becoming symptomatic cases followed Gamma distributions, and the estimated means were 6.6 days (all cases), 5.1 days (immediately confirmed cases), and 10.4 days (later becoming symptomatic cases; Table 2). A small number of consecutive generations of patients may have had another exposure to an unidentified infection source, which couldn’t be excluded in our analysis. Thus, we performed a sensitivity analysis using different delay times between symptom onset of index patients and consecutive generations of patients. The estimated results were 6.6 to 9.3 days for all cases, 5.1 to 7.6 days for immediately confirmed cases, and 10.4 to 12.3 days for later becoming symptomatic cases (Additional file 1: Table S3). We also compared the distributions of the incubation period and the serial interval for each of these groups (Fig. 3). In each case, the serial interval was shorter than incubation period. Incubation period and serial interval of female were less than those of male (Fig. 4A). Except in the immediately confirmed cases, incubation period and serial interval of cases aged 19+ were less than those of cases aged 0–18 (Fig. 4B).

**Table 2:** Estimated incubation period and serial interval of confirmed COVID-19 patients in Shijiazhuang

Characteristic	Estimated incubation period (days)		Estimated serial interval (days)	
	Mean (95%CI)	Parameters of Weibull distribution (shape, scale)	Mean (0.025^**th**^,0.975^**th**^)	Parameters of gamma distribution (shape, rate)
**All**	11.6 (10.6, 12.7)	2.75, 13.07	6.6 (0.6, 20.0)	1.63, 0.25
**Immediately confirmed cases**	10.6 (9.5, 11.7)	2.82, 11.89	5.1 (0.5, 15.0)	1.76, 0.34
**Later becoming symptomatic cases**	15.8 (13.7, 17.8)	4.35, 17.29	10.4 (1.7, 26.6)	2.50, 0.24

**Fig. 5:**
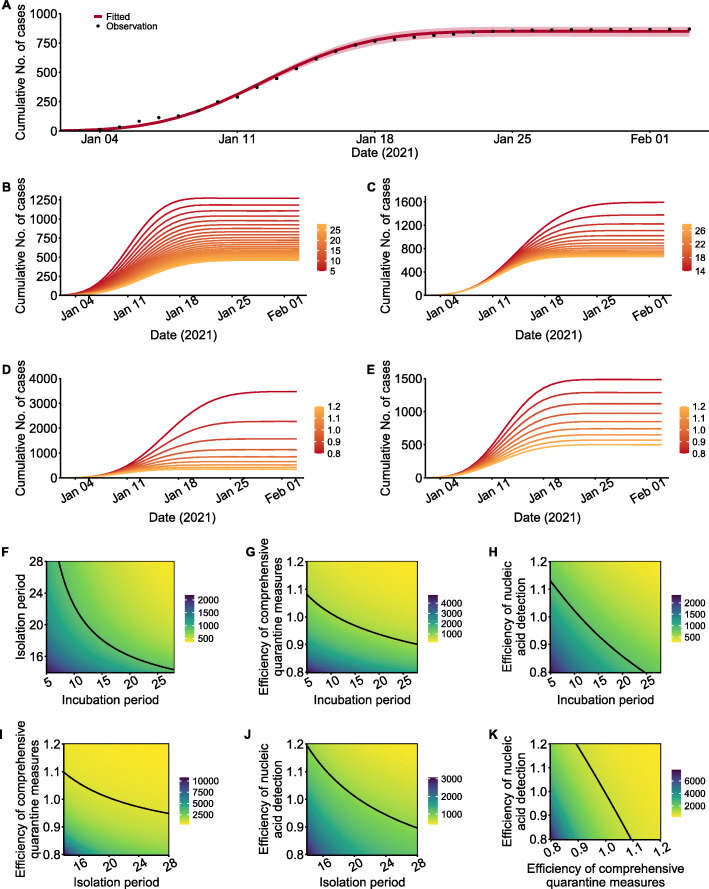
Model fitting results and model predictions of the cumulative number of cases under different scenarios. Model results (red line) and observations (black dots; **A**). Cumulative number of cases resulting from different incubation periods (**B**, range 5–25 days), isolation times (**C**, range 14–28 days), effectiveness of comprehensive quarantine measures (**D**, range 0.8–1.2 times of the initial values), and effectiveness of nucleic acid testing (**E**, range 0.8–1.2 times of the initial values). **F–K** show the cumulative number of cases under different combination of incubation period (5–28 days), isolation period (14–28 days) and effectiveness of comprehensive quarantine measures (0.8–1.2 times of the initial values) and effectiveness of nucleic acid detection (0.8–1.2 times of the initial values). Solid black lines in **F**–**K** are isopleths corresponding to 869 cases

The SEIR^+q^ model provided a good fit to the data (Fig. 5A). We used the fitted model to simulate the effects of changes of four key parameters (*Q*, *α*, *ω*, *ρ*_E_) that correspond to different interventions. The range of the incubation period (1/*α*) was 5 to 28 days, the range of the isolation period (1/ω) was 14 to 28 days, and the ranges of *Q* (effectiveness of comprehensive quarantine measures) and *ρ*_E_ (effectiveness of nucleic acid detection) were 0.8 times to 1.2 times their initial values. Due to the reduced infectiousness of presymptomatic and asymptomatic individuals [17, 18], use of the same prevention and control measures but a longer incubation period, the cumulative number of cases reduced but the duration of the epidemic increased (Fig. 4B, Additional file 1: Table S4). If the isolation period and the effectiveness of the comprehensive quarantine measures decreased, the cumulative number of cases increased and the epidemic period was prolonged (Fig. 5C, D; Additional file 1: Table S4). Increasing the effectiveness of the nucleic acid test reduced the cumulative number of cases, but had little effect on the duration of the epidemic (Fig. 5E, Additional file 1: Table S4).

If the incubation period was shorter, more stringent prevention and control measures were needed to curb the epidemic and minimize the cumulative number of cases (Fig. 5F–H). A comparison of the effect of the three measures under the same change of incubation period indicated that strengthening comprehensive quarantine measures provided the most effective intervention, followed by improving nucleic acid testing; extending the isolation period had limited effect. Thus, if the incubation period was less than 7.5 days, more than 28 days of quarantine/isolation were needed to control the outbreak (Fig. 5F). If the isolation time decreased, more stringent quarantine measures and nucleic acid testing were needed to control the outbreak (Fig. 5I, J). To minimize the cumulative number of cases, more improvements were needed for nucleic acid testing than quarantine. If the effectiveness of the comprehensive quarantine measures declined by 10%, a 20% or more increase in the effectiveness of nucleic acid testing was needed to minimize the cumulative number of cases (Fig. 5K).

## Discussion

We identified 869 patients with confirmed symptomatic COVID-19 and found a higher proportion of females than males, consistent with previous studies [4, 21, 22]. In comparison with previous studies, our COVID-19 patients were younger, with a higher proportion of patients aged 0 to 19 years old. Some studies suggested that the risk of infection from family members was greater than that from other contacts [23–25]. Therefore, after closure of schools on 6 January, children and adolescents may have had a greater risk of infection, and this might have contributed to the relatively high proportion of young patients. We also found that the later becoming symptomatic cases were younger than the immediately confirmed cases, meaning that symptoms might be milder or appear later in children and young people than in adults [26, 27]. However, there was no significant difference in the percentages of later becoming symptomatic cases and immediately confirmed cases among males and females.

We estimated that the mean incubation period was 11.6 days, longer than reported in previous studies [1, 2, 9, 10, 28]. Obviously, the mean incubation period of later becoming symptomatic cases (15.8 days) was much longer than that of immediately confirmed cases (10.6 days). The mean serial interval was 6.6 days, shorter than the serial interval (7.5 days) during the first COVID-19 epidemic wave in Wuhan [1], but longer than other studies [9, 29, 30]. And the serial interval of our later becoming symptomatic cases was much longer (10.4 days). There were also several studies reported that the incubation periods of cases associated with the Wuhan epidemic were longer than 14 days [31, 32]. Besides, taking Shanghai as another example, based on Zhang. et al.’s work [9] with averaged incubation period 5.2 days, we estimate that the average number of patients’ incubation period larger than 14-day centralized quarantine should be around only one among 2124 imported cases as of 4 September 2021 [33]. However, more than five onset COVID-19 cases after 14-day centralized quarantine have been reported in the last 1.5 years in Shanghai. In addition, other cities, such as Guangzhou, Beijing, and Chengdu, also reported several import cases that onset after 14-day centralized quarantine [34]. Besides, there were increasingly number of imported cases in the winter period who were diagnosed with COVID-19 after lifting of the 14-day centralized quarantine in China [34]. Those facts suggested the possibility of a prolonged incubation period.

There have been evidence that longer incubation period of SARS-CoV-2 corresponded to less severe symptoms [35–37], and the young age of host might also have influences on the longer incubation period [38]. A majority of patients in this study were young, which might present with mild symptoms, a fact that may lead to the longer incubation period. Besides, increased virus mutations may have contributed to the longer incubation period in our patients. The mutation of virus, such as higher number of protein-coding genes or GC content (namely, contents of Guanine (G) and Cytosine (C) in viral RNA), corresponds to a longer incubation time [35]. Higher GC content leads to stable secondary structures in the virus RNA, which means that the ribosome needs to disrupt higher kinetic barriers during translation and needs longer translation time [39]. The longer cumulative translation time extends the replication cycle of virus and finally results the longer incubation period [35].

A longer incubation period indicates the need for a longer centralized quarantine period to identify infected individuals and control the outbreak. The large number of covert cases and the high transmissibility of SARS-CoV-2 are two key features of COVID-19 outbreaks [40], and a longer incubation period exacerbates these features. A longer incubation period makes it more difficult to identify infected individuals, and a longer infectious period may lead to infection of more people before diagnosis or quarantine. The incubation period was much longer than serial interval, indicating that mass transmission might occur before symptom onset. In rural areas, routine surveillance and prevention procedures are more difficult [41, 42], and this may lead to failure in the timely detection of new cases. The first COVID-19 case in the Shijiazhuang outbreak was reported on 2 January 2021; however, 196 (22.6%) infected individuals were identified by close contact tracking by 6 January. This indicates that the virus had already spread in the population before the first case was identified, presumably because of the long incubation period.

Due to the timely response in Shijiazhuang, including the rapid implementation of quarantine and lockdown, most cases of COVID-19 were in Gaocheng District and there were no cases related to this outbreak in other provinces of China. Moreover, because of the massive use of city-wide nucleic acid testing, many infected people were found before symptom onset and were isolated before January 15. These interventions had a significant impact on controlling the transmission and spread of the virus.

The results of our SEIR^+q^ model showed that quarantine measures, which included close contact tracking, centralized isolation, home quarantine, and distant centralized quarantine, had a more significant effect on the outbreak than massive nucleic acid testing. Our model also indicated that if the comprehensive quarantine intensity was reduced by 10%, then at least 20% or more nucleic acid tests were needed to minimize the cumulative number of cases. If so, this would lead to additional expenditures of $18 million, because nucleic acid testing costs $3 per sample [43]. Although nucleic acid testing played a significant role in identification of cases, especially asymptomatic and presymptomatic cases in the community, the possibility of false-negatives [44] and the delay in receiving the results [45] are two main limitations. Due to false-negative results, some asymptomatic or presymptomatic individuals who are infected may not be isolated and might infect others [44]. To avoid the false-negative results, it is usually necessary to test the same person several times, which needs intensive manpower and resources and depends on the economic strength of the country/city. Thus, repeat testing is not applicable in all cities. Therefore, efforts are needed to improve the sensitivity of the nucleic acid testing, and standard sampling and preservation of samples are also needed to reduce the false-negative results. In addition, the effects of nucleic acid testing and massive quarantine rely on cooperation and compliance of the general population, so education of the public on these important interventions is also important.

This study had limitations. First, there was no detailed information (exposure interval, date of symptom onset, etc.) of individuals with asymptomatic infections, nor was there information on clinical severity of symptomatic cases, which might have biased our estimates of the incubation period and serial interval. Besides, incubation period might be overestimated, due to the cases may have multiple contacts with index cases and the exact dates of exposure were unknown. Although it is worthwhile to further study, however, a number of published papers have clarified that the incubation period of asymptomatic patients is longer that of symptomatic patients [31, 46]. Second, in the clusters used to estimate serial interval, infectors of some cases (especially those who develop symptoms 1 to 3 days after the date of symptom onset of the index cases) might be unidentified. Sensitivity analysis with censoring cluster data [9] have been conducted to overcome the limitation. Further, our study has similar limitations as other studies using SEIR model. For example, we do not consider the heterogeneity of the population and the randomness of infection in the SEIR^+q^ model. Age stratified SEIR model or agent-based model would be preferable to analyse the characteristics of the outbreak and simulate more real-world interventions, which also require individual information in more details.

## Conclusions

In conclusion, our study of the COVID-19 outbreak in Shijiazhuang estimated that the mean incubation period was 11.6 days. This suggests it is necessary to prolong the isolation/quarantine period currently so that all potentially infected individuals can be followed. Importantly, a long incubation period may lead to significant transmission of the virus before identification of initial cases. The serial interval (6.6 days) was substantial shorter than the incubation period, suggesting that a significant amount of secondary transmission may occur prior to illness onset. Although resource intensive, the use of comprehensive quarantine measures and mass nucleic acid testing were effective to reduce the disease transmission quickly.

## Supplementary Information

**Additional file 1: Table S1.** Meanings and initial values of parameters in the SEIR^+q^ model. **Table S2.** Mean and standard deviation (SD) of the estimated incubation period for patients with confirmed COVID-19. **Table S3.** Estimated gamma distributions of the serial interval using different delays between symptom onset of index patients and secondary patients. **Table S4.** Cumulative number of patients and the end date of the COVID-19 outbreak for different incubation periods, isolation periods, efficiency of comprehensive quarantine measures, and efficiency of nucleic acid testing.

## Data Availability

The datasets used and/or analyzed during the current study are available from the corresponding author on reasonable request.

## Abbreviations

95%CI: 95% confidence interval
COVID-19: Coronavirus disease 2019
LooIc: Leave-one-out Information Criterion
NPIs: Non-pharmaceutical interventions
SARS-CoV-2: Severe acute respiratory syndrome coronavirus 2
SEIR: Susceptible Exposed Infectious Recovered
