# Supplementary material for: Effects of prolonged incubation period and centralized quarantine on the COVID-19 outbreak in Shijiazhuang, China: a modeling study
Source: BMC Med. 2021 Dec 7;19:308. doi: 10.1186/s12916-021-02178-z (PMC8648499; doi:10.1186/s12916-021-02178-z)
Supplement: Supplementary file 1 — Additional file 1: Table S1. Meanings and initial values of parameters in the SEIR+q model. Table S2. Mean and standard deviation (SD) of the estimated incubation period for patients with confirmed COVID-19. Table S3. Estimated gamma distributions of the serial interval using different delays between symptom onset of index patients and secondary patients. Table S4. Cumulative number of patients and the end date of the COVID-19 outbreak for different incubation periods, isolation periods, efficiency of comprehensive quarantine measures, and efficiency of nucleic acid testing. [file 12916_2021_2178_MOESM1_ESM.docx]

**Additional files for**

**Effect of prolonged incubation period and centralized quarantine on the COVID-19 outbreak in Shijiazhuang, China: a model study**

Wenlong Zhu ^1^, Mengxi Zhang ^1^, Jinhua Pan ^1^, Ye Yao ^1*^, Weibing Wang ^1, 2*^

^1^ School of Public Health, Shanghai Institute of Infectious Disease and Biosecurity, Fudan University, 138 Yi Xue Yuan Road, Shanghai 200032, China

^2^ Key Laboratory of Public Health Safety of Ministry of Education, Fudan University, 138 Yi Xue Yuan Road, Shanghai 200032, China

* Corresponding authors:

Dr. Ye Yao

School of Public Health, Fudan University, 138 Yi Xue Yuan Road, Shanghai 200032, China (e-mail: yyao@fudan.edu.cn).

Dr. Weibing Wang

Department of Epidemiology, School of Public Health; Shanghai Institute of Infectious Disease and Biosecurity; Key Laboratory of Public Health Safety (Ministry of Education), Fudan University, 138 Yi Xue Yuan Road, Shanghai 200032, China (e-mail: wwb@fudan.edu.cn).

**This file includes:**

Table S1 to Table S4.

**Table S1.** Meanings and initial values of parameters in the SEIR^+q^ model.

| **Parameters** | **Meaning** | **Values** | **Source** |
| --- | --- | --- | --- |
| N | Total population of Shijiazhuang | 10 million | Ref [14] |
| S | Susceptible people | 10 million | Ref [14] |
| S_hq_ | Home quarantined susceptible people | 0 | Assumed |
| E | Exposed (infected) people | 12 | Assumed |
| E_cq_ | Centralized quarantined exposed (infected) people | 0 | Assumed |
| I | Infectious (symptomatic) people | 1 | Assumed |
| I_q_ | Isolated (confirmed) infectious (symptomatic) people | 1 | Ref [8] |
| R | Removed (recovered or death) | 0 | Assumed |
| Q | The number of comprehensive quarantined persons per day | 1.1 million | Estimated |
| ω | The rate of release from isolation (the reciprocal of the isolation period) | 1/21 | Ref [15] |
| ρ_E_ | The probability of an exposed person being found and centralized quarantine | 415/869 | Computed |
| β | The average number of infected individuals per day | 2.893 | Estimated |
| ε | The transmission coefficient of exposed people (compared with symptomatic people) | 0.5 | Ref [16-18] |
| α | The transition rate from latent infection to symptomatic infection (the reciprocal of the incubation period) | 1/11.6 | Computed |
| η | The isolation rate of symptomatic people | 1/2 | Assumed |
| γ | The transition rate from disease confirmation to recovery or death | 1/12 | Ref [19] |

**Table S2.** Mean and standard deviation (SD) of the estimated incubation period for patients with confirmed COVID-19.

|  | **No. of cases** | **Distribution** | **Estimated incubation period (days)** | | | **Parameters** |
| --- | --- | --- | --- | --- | --- | --- |
|  |  |  | **Mean (95%CI)** | **SD (95%)** | **LooIC** |  |
| **All** | 113 | Weibull | 11.6 (10.6, 12.7) | 4.6 (3.9, 5.4) | **693.3** | shape=2.75, scale=13.07 |
|  |  | Gamma | 11.5 (10.4, 12.6) | 4.8 (4.1, 5.8) | 699.6 | alpha=5.63, beta=0.49 |
|  |  | Lognormal | 11.5 (10.3, 12.7) | 5.6 (4.5, 7.2) | 705.7 | mu=2.33, sigma=0.46 |
| **Immediately confirmed cases** | 91 | Weibull | 10.6 (9.5, 11.7) | 4.1 (3.4, 4.9) | **543.1** | shape=2.82, scale=11.89 |
|  |  | Gamma | 10.4 (9.3, 11.5) | 4.3 (3.5, 5.4) | 548.7 | alpha=3.77, beta=0.55 |
|  |  | Lognormal | 10.3 (9.2, 11.7) | 5.1 (4.0, 6.8) | 553.8 | mu=2.23, sigma=0.46 |
| **Later becoming symptomatic cases** | 22 | Weibull | 15.8 (13.7, 17.8) | 4.1 (2.8, 6.2) | **136.0** | shape=4.35, scale=17.29 |
|  |  | Gamma | 15.7 (13.9, 17.6) | 3.6 (2.5, 5.7) | 137.3 | alpha=18.48, beta=1.17 |
|  |  | Lognormal | 15.8 (13.6, 18.6) | 4.8 (3.1, 8.7) | 137.9 | mu=2.71, sigma=0.30 |

**Table S3.** Estimated gamma distributions of the serial interval using different delays between symptom onset of index patients and secondary patients.

|  | **Delay between date of symptom onset of index and secondary cases** | **Number of clusters** | **No. of observations (excluding index cases)** | **Serial interval (0.025^th^, 0.975^th^), empirical data** | **Serial interval (0.025^th^, 0.975^th^), estimated from the fit of a gamma distribution** | **Parameters of the gamma distribution (shape, rate)** |
| --- | --- | --- | --- | --- | --- | --- |
| **All** | > 0 days | 64 | 74 | 6.6 days, (1, 17.3.0) | 6.6 days, (0.6, 20.0) | 1.63, 0.25 |
|  | > 1 day | 56 | 65 | 7.4 days, (2.0, 17.8) | 7.4 days, (1.1, 19.6) | 2.30, 0.31 |
|  | > 2 days | 48 | 55 | 8.4 days, (3.0, 18.3) | 8.4 days, (1.9, 19.7) | 3.24, 0.39 |
|  | > 3 days | 41 | 47 | 9.3 days, (4.0, 18.7) | 9.3 days, (2.7, 19.8) | 4.37, 0.47 |
| **Immediately confirmed cases** | > 0 days | 46 | 53 | 5.1 days, (1.0, 16.4) | 5.1 days, (0.5, 15.0) | 1.76, 0.34 |
|  | > 1 day | 38 | 44 | 6.0 days, (2.0, 16.8) | 6.0 days, (1.1, 14.8) | 2.77, 0.46 |
|  | > 2 days | 32 | 37 | 6.7 days, (3.0, 17.0) | 6.7 days, (1.7, 15.0) | 3.77, 0.56 |
|  | > 3 days | 26 | 30 | 7.6 days, (4.0, 17.0) | 7.6 days, (2.5, 15.6) | 4.97, 0.65 |
| **Later becoming symptomatic cases** | > 0 days | 20 | 21 | 10.4 days, (2.0, 19.0) | 10.4 days, (1.7, 26.6) | 2.50, 0.24 |
|  | > 1 day | 20 | 21 | 10.4 days, (2.0, 19.0) | 10.4 days, (1.7, 26.6) | 2.50, 0.24 |
|  | > 2 days | 17 | 18 | 11.8 days, (4.3, 19.0) | 11.8 days, (3.9, 23.9) | 5.15, 0.44 |
|  | > 3 days | 16 | 17 | 12.3 days, (6.0, 19.0) | 12.3 days, (5.1, 22.6) | 7.44, 0.61 |

**Table S4.** Cumulative number of patients and the end date of the COVID-19 outbreak for different incubation periods, isolation periods, efficiency of comprehensive quarantine measures, and efficiency of nucleic acid testing.

| **Variables**  **Value** | **Cumulative No. of cases** | **End date of the outbreak**  **(2021)** |
| --- | --- | --- |
| **Incubation period** |  |  |
| 5 days | 1274 | 22 Jan |
| 6 days | 1185 | 23 Jan |
| 7 days | 1108 | 24 Jan |
| 8 days | 1040 | 24 Jan |
| 9 days | 980 | 25 Jan |
| 10 days | 925 | 25 Jan |
| 11 days | 877 | 26 Jan |
| 12 days | 833 | 26 Jan |
| 13 days | 794 | 27 Jan |
| 14 days | 757 | 27 Jan |
| 15 days | 724 | 28 Jan |
| 16 days | 694 | 28 Jan |
| 17 days | 666 | 28 Jan |
| 18 days | 640 | 28 Jan |
| 19 days | 617 | 28 Jan |
| 20 days | 594 | 29 Jan |
| 21 days | 574 | 29 Jan |
| 22 days | 555 | 29 Jan |
| 23 days | 536 | 29 Jan |
| 24 days | 520 | 29 Jan |
| 25 days | 504 | 29 Jan |
| 26 days | 489 | 29 Jan |
| 27 days | 475 | 29 Jan |
| 28 days | 461 | 29 Jan |
| **Isolation period** |  |  |
| 14 days | 1592 | 3 Feb |
| 15 days | 1377 | 1 Feb |
| 16 days | 1225 | 30 Jan |
| 17 days | 1111 | 29 Jan |
| 18 days | 1023 | 28 Jan |
| 19 days | 953 | 27 Jan |
| 20 days | 897 | 27 Jan |
| 21 days | 850 | 26 Jan |
| 22 days | 811 | 26 Jan |
| 23 days | 778 | 25 Jan |
| 24 days | 749 | 25 Jan |
| 25 days | 724 | 25 Jan |
| 26 days | 702 | 25 Jan |
| 27 days | 683 | 24 Jan |
| 28 days | 666 | 24 Jan |
| **Efficiency of comprehensive quarantine measures^§^** |  |  |
| 0.80 | 3476 | 3 Feb |
| 0.85 | 2270 | 1 Feb |
| 0.90 | 1569 | 30 Jan |
| 0.95 | 1134 | 28 Jan |
| 1.00 | 850 | 26 Jan |
| 1.05 | 657 | 25 Jan |
| 1.10 | 521 | 24 Jan |
| 1.15 | 422 | 23 Jan |
| 1.20 | 349 | 22 Jan |
| **Efficiency of nucleic acid detection^§^** |  |  |
| 0.80 | 1486 | 26 Jan |
| 0.85 | 1289 | 26 Jan |
| 0.90 | 1120 | 26 Jan |
| 0.95 | 975 | 26 Jan |
| 1.00 | 850 | 26 Jan |
| 1.05 | 743 | 26 Jan |
| 1.10 | 650 | 26 Jan |
| 1.15 | 570 | 26 Jan |
| 1.20 | 502 | 26 Jan |

§ The comprehensive quarantine measures, which mainly influenced the number of isolated susceptible people (Q), included close contact tracking, home quarantine, and similar quarantine measures. The efficiency of nucleic acid testing, which had significant impact on the rate of centralized isolation of exposed people (ρ_E_), consisted of the scale and speed of the test. The Q and ρ_E_ values ranged from 0.80 to 1.20-times that of the initial values.
